# Association of patient socioeconomic status with outcomes after palliative treatment for disseminated cancer

**DOI:** 10.1002/cam4.7028

**Published:** 2024-05-06

**Authors:** Richard C. Maduka, Maureen E. Canavan, Samantha L. Walters, Theresa Ermer, Peter L. Zhan, Michael F. Kaminski, Andrew X. Li, Matthew D. Pichert, Michelle C. Salazar, Elizabeth H. Prsic, Daniel J. Boffa

**Affiliations:** ^1^ Division of Thoracic Surgery, Department of Surgery Yale University School of Medicine New Haven Connecticut USA; ^2^ Yale Cancer Center Advanced Training Program for Physician Scientist, NIH T32 Fellowship Yale University School of Medicine New Haven Connecticut USA; ^3^ Cancer Outcomes Public Policy and Effectiveness Research (COPPER) Center, Department of Internal Medicine Yale University School of Medicine New Haven Connecticut USA; ^4^ Faculty of Medicine Friedrich‐Alexander‐University Erlangen‐Nürnberg Erlangen Germany; ^5^ London School of Hygiene & Tropical Medicine University of London London UK; ^6^ National Clinician Scholars Program Yale University School of Medicine New Haven Connecticut USA; ^7^ Palliative Care Program, Department of Internal Medicine Yale School of Medicine New Haven Connecticut USA

**Keywords:** cancer education, cancer management, chemotherapy, palliative treatment

## Abstract

**Background:**

Palliative treatment has been associated with improved quality of life and survival for a wide variety of metastatic cancers. However, it is unclear whether the benefits of palliative treatment are uniformly experienced across the US cancer population. We evaluated patterns and outcomes of palliative treatment based on socioeconomic, sociodemographic and treating facility characteristics.

**Methods:**

Patients diagnosed between 2008 and 2019 with Stage IV primary cancer of nine organ sites were analyzed in the National Cancer Database. The association between identified variables, and outcomes concerning the administration of palliative treatment were analyzed with multivariable logistic regression and Cox proportional hazard models.

**Results:**

Overall 238,995 (23.6%) of Stage IV patients received palliative treatment, which increased over time for all cancers (from 20.7% in 2008 to 25.6% in 2019). Palliative treatment utilization differed significantly by region (West less than Northeast, OR: 0.55 [0.54–0.56], *p* < 0.001) and insurance payer status (uninsured greater than private insurance, OR: 1.35 [1.32–1.39], *p* < 0.001). Black race and Hispanic ethnicity were also associated with lower rates of palliative treatment compared to White and non‐Hispanics respectively (OR for Blacks: 0.91 [0.90–0.93], *p* < 0.001 and OR for Hispanics: 0.79 [0.77–0.81] *p* < 0.001).

**Conclusions:**

There are important differences in the utilization of palliative treatment across different populations in the United States. A better understanding of variability in palliative treatment use and outcomes may identify opportunities to improve informed decision making and optimize quality of care at the end‐of‐life.

## BLURB FOR ETOC

As stated in the manuscript, this study uses de‐identified data from human participants within the National Cancer Database, and the study has been approved by the Yale School of Medicine Institutional Review Board with consent waived.

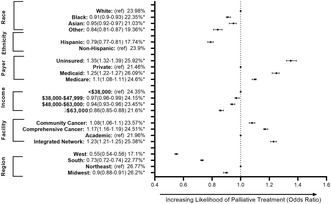

## 1 INTRODUCTION

Despite impressive innovations in cancer treatment, many cancer patients present with or progress to incurable disease. The treatment strategy in this setting is trending toward interventions designed to improve quality of life, which includes palliative care.^1^, ^2^, ^3^ The concept of palliative care has evolved tremendously over the past several decades. Palliation has proven to be effective in addressing a wide range of needs for patients with metastatic cancer and is implemented in a variety of delivery models including outpatient palliative care clinics, inpatient palliative care consultation teams, acute palliative care units (APCUs), community based palliative care, and hospice care. Most models have been associated with improved patient outcomes including quality of life and improving symptom burden.^4^ Additionally, inpatient palliative care consultation teams, which are present in approximately 90% of NCI‐designated cancer centers,^5^, ^6^ have also been noted to have a positive impact on cost of care.^7^, ^8^, ^9^, ^10^ However, it is important to note that the administration of palliative care is also associated with challenges including managing misconceptions for both patients and providers,^11^ more treatment options, and challenges with prognostication and coordination of care.

Coordinated patient care that addresses physical as well as psychosocial issues (including pain control, depression, disruption of family life, and financial concerns), can result in improved overall satisfaction with treatment outcomes and reduced cost of care. Therefore, it is unsurprising that palliative care provision has been associated with improved patient quality of life.^1^, ^2^, ^12^, ^13^ For several tumor types, early integration of palliative care has also been associated with an improved survival.^1^, ^3^, ^12^, ^14^, ^15^ Subsequently, integration of palliative care is the standard of care for metastatic patients.

For patients diagnosed with Stage IV, the goal of treatment can shift to comfort‐directed care and addressing physical functioning and addressing symptom burden.^16^, ^17^, ^18^ This initial first‐line therapy for these patients can be provided by the oncologist. Palliative treatment as a subset of overall palliative care can specifically focus on tumor directed therapy to improve the patient well‐being using strategies that decrease tumor burden (surgery, radiation, and systemic therapy).^19^ Depending on the individual cancer type, these modalities are used at different rates^20^, ^21^, ^22^, ^23^ but surgical treatment, systemic therapy, and radiation as well as pain management are all included as first‐line treatments for advanced and metastatic patients.^19^, ^24^, ^25^

While there is variation across tumor types, it has been estimated that only 10%–30% of eligible patients with cancer receive palliative treatment including treatments for pain management and tumor directed therapy in the United States.^26^, ^27^, ^28^, ^29^, ^30^ Furthermore, this care is not uniform across all patient populations. Concerns have been raised regarding the access to palliative treatment by socioeconomically disadvantaged patient populations.^31^, ^32^, ^33^, ^34^, ^35^, ^36^, ^37^, ^38^, ^39^, ^40^, ^41^, ^42^ As we attempt to understand the landscape for palliative treatment options it is important to understand the patterns of utilization to address any inequities that might exist, specifically those related to sociodemographic factors as they can serve as potential levers for interventions.

The National Cancer Database (NCDB) captures first‐line treatment for approximately 70% of cancer patients in the United States, including palliative care.^43^ We examined the patterns of palliative treatment across multiple tumor types, in hopes to identify opportunities to facilitate a more optimal delivery of palliative care in the United States.

## 2 METHODS

### 2.1 Data source

The NCDB is a joint project of the American Cancer Society and the American College of Surgeons, which captures incident cancer cases diagnosed or treated at over 1500 Commission on Cancer accredited facilities.^43^ The NCDB captures 72% of all newly diagnosed malignancies in the United States annually.^44^ Overall coverage of cancer cases in the NCDB has remained relatively stable with a slight increase from 67% observed in 2004–2006. Case coverage also increased slightly between 2012 and 2014, as did the number of Commission on Cancer‐accredited facilities, which increased from 1455 to 1475 and represents approximately 25% of acute‐care facilities.^44^

De‐identified NCDB data were analyzed in accordance with our research protocol, which was approved by the Yale School of Medicine institutional review board with consent waived.

### 2.2 Patient selection

The NCDB 2020 Participant User File (PUF) was queried for adult patients (≥18 years old) diagnosed with Stage IV cancer originating within lung (non‐small cell lung cancer [NSCLC]), pancreas, colon, breast, prostate, kidney, stomach, esophagus, and rectum between 2008 and 2019. These cancer sites were selected because: (1) based on our preliminary analysis across all tumor types in the NCDB, case counts for patients receiving palliative treatment were among the highest for these tumor types, (2) they represented different anatomic regions, impairing quality of life in different ways, and (3) their management typically involves different multidisciplinary care teams.

Patients with missing data for vital status, follow‐up time, or time to treatment were excluded. Additionally, only patients who had documented follow up of at least 6 months (or died within that time frame) were included (Consort Diagram: Figure S1). Overall, 1,013,148 Stage IV patients were included. A sensitivity analysis of excluded patients failed to identify any obviously important differences to the primary study group.

### 2.3 Covariates and measured outcomes

Independent variables included cancer site, insurance status, age (i.e., <50, 50–59, 60–69, 70–79, ≥80), sex, year of cancer diagnosis, race, Hispanic ethnicity, median income for the zip code of the patient's residence, modified Charlson–Deyo comorbidity index (CD score) (i.e., 0, 1, 2, ≥3), facility type (i.e., community cancer program, comprehensive community cancer program, academic program, and integrated network program), and region (based on US census regions. i.e., Midwest, West, Southeast, and South).

The primary outcome was administration of any care provided with the intent to palliate or alleviate symptoms which we collapsed into a binary (yes/no) response. Palliative treatment is defined in the NCDB as a multilevel categorical variable with the following response options: no palliative treatment, surgery, radiation therapy, systemic therapy (chemotherapy, hormone therapy, or other systemic drugs), other pain management therapy, a combination of treatments without pain management therapy, and a combination of palliative treatment options with pain management therapy.

We identified the exact treatment type or a combination of these modalities administered.

We also assessed secondary outcomes including time to palliative treatment, defined as the number of days between the initial cancer diagnosis and initiation of first treatment, and treatment refusal. Within the NCDB, patients were classified as refusing treatment for a treatment modality (surgery, radiation, systemic, other therapy, or transplant) if the patient refused that recommended treatment modality, made a blanket refusal of all recommended treatment, or refused all treatment before any was recommended. We classified a patient as refusing treatment if they made any type of refusal.

### 2.4 Statistical analysis

Differences in patient characteristics of those who received palliative treatment compared with those who did not were analyzed using chi‐squared tests for categorical variables and *t*‐tests for continuous variables.

To identify factors independently associated with receipt of palliative treatment, multivariable logistic regression models were created for all nine cancers combined and each individual cancer separately. Additionally, we also conducted multinomial regression for each individual treatment modality with palliative intent (surgery, radiation, systemic therapy, pain management only and any combination of treatment modalities) compared with no palliative treatment.

For secondary analysis, Cox proportional hazards models were used to identify factors associated with differences in time to palliative treatment initiation from Stage IV diagnosis and multivariable logistic regression models were created to identify factors independently associated with treatment refusal for the overall sample.

All analysis models were adjusted for cancer type, year of diagnosis, age at diagnosis, CD score, sex, race, ethnicity, insurance status, median income, treatment facility type and region. These included variables have been associated with receipt of palliative treatment and were significant in unadjusted analysis. Due to the large number of statistical tests performed we applied a Bonferroni correction to our models to account for multiple testing^45^ (for each model there were 39 tests performed, therefore statistical significance was achieved when the two‐sided *p* < 0.001 (0.05/39 = 0.0013)).

Satisfaction of the proportional hazards' assumption was tested using the Martingale residuals in the adjusted Cox models. No violations of the proportional hazards' assumption were identified. All data analysis was conducted with Statistical Analysis System version 9.4 (SAS Institute, Cary, NC).

### 2.5 Missing data strategy

For all variables, data were missing in less than 13%. Complete case analysis with a missing category was used for region, facility type (these variables were systematically suppressed for patients younger than 40 and thus missing not at random) and our outcome variables. All other variables appeared to be missing at random and we used a multiple imputation approach to address missing data in our models. Specifically, we used the fully conditional method (FCS) with a generalized logit distribution and specified the “classeffects = option” statement to allow for non‐ordered distribution of categorical predictors in our imputation model. We included the following variables within each cancer specific imputation model: year of diagnosis, age at diagnosis, CD score, sex, race, ethnicity, insurance status, median income, and treatment facility type and region. We incorporated 10 imputation datasets because we saw no noticeable difference between point estimates or standard errors across imputation datasets for our imputed variables at this level.

## 3 RESULTS

### 3.1 Patient demographics

Overall, 1,013,148 eligible patients with Stage IV cancer from one of the nine selected primary sites were selected, including 238,995 (23.6%) who received palliative treatment (Table 1). The largest subgroup of patients receiving palliative treatment had non‐small cell lung cancer (*n* = 125,293, 52.4% of total), while the smallest subgroup had rectal cancer (*n* = 6367, 2.7% of total). The median age across all 9 cancers was 65 (IQR 57–74) years. Patients receiving palliative treatment were most likely to be male (55.1%), White (83.0%), non‐Hispanic (96.2%), and have Medicare insurance (51.8%).

**TABLE 1:** Association of covariates with receipt of any palliative treatment.

	Palliation	Row %	No Palliation	Row %	*p*‐value
Cancer site					
Breast	21,610	22.85	72,960	77.15	<0.001
Colon	17,808	13.13	117,792	86.87	
Esophagus	9873	29.51	23,586	70.49	
Gastric	9772	22.56	33,547	77.44	
Kidney	11,699	20.6	45,104	79.4	
Lung	125,293	30.93	279,806	69.07	
Pancreas	22,826	23.48	74,374	76.52	
Prostate	13,747	12.24	98,558	87.76	
Rectum	6367	18.3	28,426	81.7	
Year of diagnosis					
2008	14,069	20.65	54,074	79.35	<0.001
2009	14,143	20.14	56,070	79.86	
2010	15,498	21.35	57,105	78.65	
2011	16,047	21.66	58,030	78.34	
2012	17,481	22.84	59,055	77.16	
2013	19,889	24.16	62,432	75.84	
2014	21,503	25.04	64,356	74.96	
2015	22,782	25.18	67,682	74.82	
2016	23,979	25.14	71,415	74.86	
2017	24,104	24.36	74,851	75.64	
2018	24,039	24.29	74,940	75.71	
2019	25,461	25.56	74,143	74.44	
Age					
<50	20,324	20.77	77,523	79.23	<0.001
50–59	51,789	23.71	166,607	76.29	
60–69	75,783	23.65	244,680	76.35	
70–79	61,752	24.24	193,005	75.76	
≥80	29,347	24.12	92,338	75.88	
CD score					
0	158,221	22.28	552,083	77.72	<0.001
1	51,965	25.74	149,891	74.26	
2	18,041	28.27	45,785	71.73	
3 or more	10,768	28.98	26,394	71.02	
Sex					
Male	131,827	23.1	438,902	76.9	<0.001
Female	107,168	24.22	335,251	75.78	
Race					
White	198,250	23.98	628,380	76.02	<0.001
Black	30,237	22.35	105,024	77.65	
Asian	7349	21.03	27,595	78.97	
Other	3159	19.36	13,154	80.64	
Ethnicity					
Non‐Hispanic	229,840	23.9	731,707	76.1	<0.001
Hispanic	9155	17.74	42,446	82.26	
Payer status					
Uninsured	10,418	25.92	29,774	74.08	<0.001
Private insurance	75,551	21.46	276,423	78.54	
Medicaid	22,815	26.09	64,644	73.91	
Medicare	123,759	24.6	379,365	75.4	
Other government	3878	25.88	11,105	74.12	
Status unknown	2574	16.7	12,842	83.3	
Median income					
<$38,000	40,411	24.35	125,547	75.65	<0.001
$38,000–$47,999	51,067	24.15	160,380	75.85	
$48,000–$63,000	56,541	23.45	184,609	76.55	
≥$63,000	59,647	21.6	216,546	78.4	
Unknown	31,329	26.46	87,071	73.54	
Facility type					
Community program	17,599	23.57	57,063	76.43	<0.001
Comprehensive program	92,279	24.51	284,221	75.49	
Academic program	76,017	21.96	270,166	78.04	
Integrated network	48,836	25.38	143,557	74.62	
Unknown	4264	18.21	19,146	81.79	
Region					
Northeast	57,107	26.77	156,246	73.23	<0.001
Midwest	69,784	26.2	196,552	73.8	
South	82,812	22.77	280,837	77.23	
West	25,028	17.1	121,372	82.9	
Unknown	4264	18.21	19,146	81.79	
Total	238,995		774,153		

**FIGURE 1:**
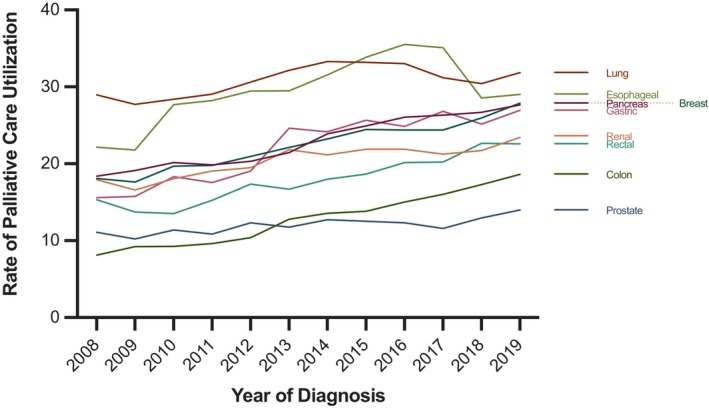
Rate of palliative treatment over time among nine solid tumor types.

The rates of palliative treatment differed by tumor type and increased over time (Figure 1). For example, in 2019, a total of 18.6% of patients with colon cancer received palliative care, compared to 31.9% of patients with lung cancer. Over time, palliative treatment increased at different rates across the different tumor types. Only a modest increase in palliative treatment was seen in the prostate cancer group (11.1% in 2008 vs. 14.0% in 2019), while the utilization among patients with gastric cancer increased from 15.6% to 26.9% during the same period.

### 3.2 Predictors of palliative treatment receipt

**FIGURE 2:**
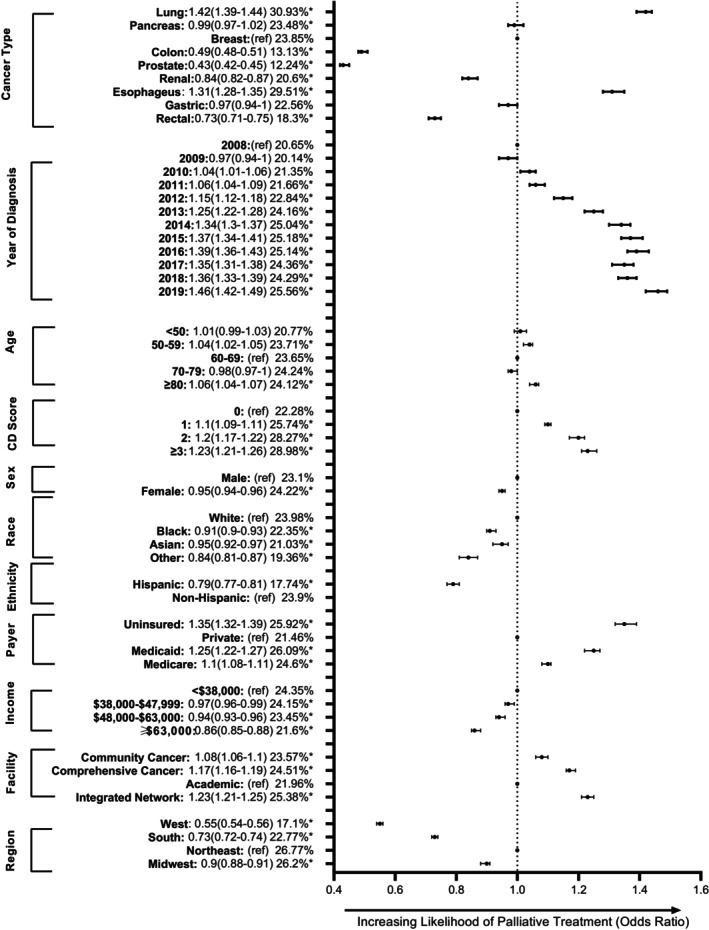
Forest plot for the odds of palliative treatment.

A multivariable logistic regression model (across all nine cancers in aggregate) was performed and identified multiple predictors of palliative treatment receipt that remained significant after applying a Bonferroni correction with a threshold of 0.001 (Figure 2). Primary tumor site was associated with receipt of palliative treatment. For example, patients with NSCLC were more likely to receive palliative treatment than patients with breast cancer (OR: 1.42 [1.39–1.44], *p* < 0.001). Sociodemographic and health predictors were identified. For example, a CD score ≥3 was associated with an increased receipt of palliative treatment compared to a CD score of 0 (OR: 1.23 [1.21–1.26], *p* < 0.001). Black race and Hispanic ethnicity were both associated with lower rates of palliative treatment compared to White and non‐Hispanics respectively (OR for Blacks: 0.91 [0.90–0.93], *p* < 0.001 and OR for Hispanics: 0.79 [0.77–0.81] *p* < 0.001).

Socioeconomic predictors were evaluated. Uninsured patients were more likely to receive palliative treatment than those with private insurance (OR: 1.35 [1.32–1.39], *p* < 0.001). The highest income cohort of patients (≥$63,000) were less likely to receive palliative treatment compared to the cohort with income <38,000 (OR: 0.86 [0.85–0.88], *p* < 0.001).

Finally, we observed differences in the likelihood of receiving palliative treatment by facility characteristics. Patients treated at community cancer programs were more likely to utilize palliative treatment compared with academic hospitals (OR: 1.08 [1.06–1.10], *p* < 0.001). Furthermore, patients treated at facilities located in the West were less likely to get palliative care compared with patients from the Northeast. (OR: 0.55 [0.54–0.56], *p* < 0.001).

Additionally, we also identified cancer site‐specific factors associated with palliative care administration using stratified models (Tables S3–S11). Many predictors were consistent across multiple cancer types. For example, treatment in the Northeast region of the United States led to increased receipt of palliative care across all nine tumor types. This was also true for age ≥ 80, uninsured payer status, cancer diagnosis in 2019, and a CD score of ≥3. However, several predictors differed across tumor types. Among NSCLC patients, Black race was associated with lower rates of palliative care use compared to White race patients (OR: 0.90 [0.88–0.92], *p* < 0.001), but this association was not significant for other cancer types.

Multinomial results for individual treatment modalities were largely consistent with overall receipt of palliative care among this cohort. Uninsured, Medicare, and Medicaid patients were more likely to use each treatment modality with the highest odds of palliative care receipt for pain management therapy only (Table S2). Hispanic ethnicity, Black race, higher median income and living in non‐Northeast regions were all associated with lower odds of palliative treatment receipt for each of the treatment modalities, while having higher CD scores was associated with higher odds of palliative treatment receipt for each treatment modality.

In contrast to the overall receipt of palliative care by facility type, when looking at specific type of palliative therapy, patients receiving care at community programs were less likely to receive palliative surgery (OR 0.81; 95% CI: 0.75, 0.88, *p* < 0.001) and pain management only (OR 0.76; 95% CI 0.69, 0.84, *p* < 0.001). Patients receiving care at non‐academic centers were more likely to receive palliative radiation and palliative systemic therapy compared with patients treated at academic centers. The increase in the overall receipt of palliative care by diagnosis year was most likely driven by the increase in odds of receiving palliative systemic therapy for each diagnosis year compared with 2008.

### 3.3 Time to palliative treatment initiation and patient palliative treatment refusal

A Cox proportional‐hazards model was used to identify predictors of differential treatment initiation times in patients receiving palliative therapy as their first line treatment (Figure S2). Several predictors were associated with time‐to‐treatment initiation including cancer type, patients with lung cancer had greater delay to the start of therapy when compared to patients with breast cancer (HR: 1.21 [1.19–1.23], *p* < 0.001). Younger patients (age < 50) were more likely to have a longer time from stage IV diagnosis to palliative treatment compared to patients aged 60–69 (HR: 1.11 [1.09–1.13], *p* < 0.001). Additionally, increased time to treatment initiation was seen among all other facility types compared to academic centers with the strongest difference among patients seen at integrated network cancer centers (HR: 1.30 [1.29–1.32], *p* < 0.001), while patients seen in the West had a shorter time to treatment compared to the Northeast region of the country (HR: 0.61 [0.60–0.62], *p* < 0.001).

Several predictors were associated with increased likelihood of treatment refusal. (Figure S3). Among the most notable were age ≥ 80 compared to 60–69 (OR: 1.97 [1.89–2.05], *p* < 0.001), CD score ≥3 compared to 0 (OR: 1.22 [1.15–1.30], *p* < 0.001), and having no insurance compared to private insurance (OR: 1.54 [1.44–1.65], *p* < 0.001).

## 4 DISCUSSION

Our findings suggest that the use of palliative treatment has increased over time. This is consistent with prior retrospective studies in the field.^27^, ^28^ The increased use may reflect improved awareness of the benefits of palliative treatment and a relaxation in patient eligibility, offering treatment to a broader population. Despite the overall increase in palliative treatment across cancer types, our findings suggest that palliative treatment is not uniform in the United States with several populations being less likely to receive palliative care. We also observed lower odds of receiving palliative treatment for racial and ethnic minorities, privately insured, higher income and patients seen at academic medical centers.

While several attributes predicted palliative treatment consistently across all 9 cancers studied, we found that other patterns varied across the different tumor types. One explanation could be that some attributes more closely aligned with worse overall health (i.e., older age, and higher CD index), have more predictable and homogenous social/structural effects on access to care. More complex attributes such as race and ethnicity, on the other hand, may have more heterogeneous implications for palliative treatment and palliative care receipt in general. Counterintuitively, several predictors associated with socioeconomically disadvantaged populations, such as uninsured payer status, were associated with increased use of palliative treatment. While this may represent a greater awareness and acceptance of palliative treatment among patients in these cohorts, the current literature does not support this.^46^, ^47^ A more concerning possibility is that the socioeconomically disadvantaged patients may present at the ED in higher rates with need for symptom management. Several studies have observed that lower income,^48^, ^49^ and uninsured^50^ and Medicaid patients^51^ compared with privately insured patients are more likely to present to the emergency department with complications from their cancer and thus may receive palliative treatments to relieve their symptoms at higher rates. Subsequently, as these marginalized groups have higher rates of ED use and acute care, ED visits, and hospitalizations increase toward the end of life,^52^ the need to understand and coordinate palliative care is paramount.

Our findings are largely in line with previous literature that has demonstrated lower rates of palliative treatment among racial and ethnic minorities^53^ as well as patients treated at academic medical centers.^54^ Additionally, among prostate cancer patients minoritized communities were less likely to discuss or use hospice based palliative care compared with other populations.^11^ These finding could be attributable to a combination of factors including the economics of reimbursement, inequities in treatments offered or requested, differences in communication or other structural and societal barriers to equitable access to health care at both the provider and patient level.^55^ Differences in the rates of palliative treatment between academic practices and community programs could be associated with the size and degree of overall palliative care programs and teams. Data suggest that palliative care programs are more commonly established at academic medical centers and have increasingly become a focus in medical education.^56^, ^57^ Additionally, across the United States large hospitals (those with 300 or more beds), which are disproportionately represented among academic medical centers, were seven times more likely than smaller hospitals to have palliative care available.^58^ Future research is needed to address how these levers might contribute to differences in palliative treatment patterns. Provider training and ability to initiate timely discussions regarding end‐of‐life care and decisions toward fostering positive quality‐of‐life through hospice or complementary supportive care is critical. Additionally, patient‐level attitudes and perceptions regarding the role of palliative treatment, hospice care and treatment near the end‐of‐life can also impact patient's decisions to seek palliative care and should be addressed.^53^, ^54^

We noted regional differences as well. Palliative treatment was higher in the Northeast and Midwest regions of the United States compared to the West and South. This must reflect more than socioeconomic drivers, as the concentration of disadvantaged cancer patients in the South would not be expected to be lower than the Northeast.^59^ There are quite possibly different practice philosophies that vary across regions. Palliative care legislation has grown in recent years and continues to push forward and improve treatment access. Encouragingly, the higher number of introduced and passed bills in recent years within Southern and Western states holds promise to improve awareness and equitable access to palliative care in the future.^36^, ^38^

Heterogeneity in the utilization of palliative treatment is important to understand. The variability could reflect important healthcare inequities as palliative care can be of considerable benefit to patients. However, variability in outcomes would also be important for shared decision‐making because many palliative treatments have side effects. As patients consider the risks and potential benefits of palliative treatment, a more refined understanding of their potential to extend survival and improve quality of life could impact their decision making.

Our study contains several limitations beyond those typically noted for observational research. The NCDB only captures the first course of treatment. As such, we do not have data on the considerable population of patients who first present with localized or locally advanced cancer and then progress to distant metastasis later in their disease course. Therefore, while our findings give a perspective on a large cohort of patients with advanced‐stage disease, palliative treatment patterns for patients that progress from an earlier stage cancer could differ. Additionally, the quality of race and ethnicity reporting in the NCDB is limited, as there is no standardization in the clinical setting as to how this data is collected. Prior studies have noted there are differences in the representation of certain sociodemographic strata in the NCDB. Specifically, only 50% of cancers in people of Hispanic ethnicity are captured compared with 65% in patients who are White, Black, or Asian.^43^ Therefore, it is possible that we are under capturing the utilization of palliative treatment among Hispanic patients and we can only make assumptions regarding the effect of race/ethnicity on palliative treatment utilization. An additional limitation is we were not able to assess if individual patients had support from a palliative care team or what services aside from their treatment were received and are unable to address potential issues such as goals of care planning or quality of life.

In conclusion, these data suggest heterogeneity in the administration of palliative treatment for stage IV solid tumor malignancies in the United States by sociodemographic, socioeconomic, and treatment facility characteristics. Further investigation is warranted to identify opportunities to optimize the utilization of palliative treatment and to refine prognostic estimates to enhance shared decision making.

## AUTHOR CONTRIBUTIONS

**Richard Maduka:** Conceptualization (equal); data curation (lead); formal analysis (lead); funding acquisition (lead); investigation (lead); methodology (equal); project administration (equal); software (supporting); validation (equal); visualization (equal); writing – original draft (lead); writing – review and editing (lead). **Maureen Canavan:** Data curation (equal); formal analysis (equal); investigation (equal); methodology (equal); software (lead); writing – original draft (supporting); writing – review and editing (equal). **Samantha Walters:** Conceptualization (equal); data curation (equal); formal analysis (equal); writing – review and editing (equal). **Theresa Ermer:** Conceptualization (equal); writing – review and editing (equal). **Peter Zhan:** Conceptualization (equal); investigation (equal); writing – review and editing (equal). **Michael Kaminski:** Conceptualization (equal); writing – review and editing (equal). **Andrew Li:** Conceptualization (equal); writing – review and editing (equal). **Matthew Pichert:** Conceptualization (equal); writing – review and editing (equal). **Michelle Salazar:** Conceptualization (equal); writing – review and editing (equal). **Elizabeth Prsic:** Conceptualization (equal); writing – review and editing (equal). **Daniel J. Boffa:** Conceptualization (equal); data curation (equal); formal analysis (equal); funding acquisition (equal); investigation (equal); methodology (equal); project administration (equal); resources (equal); software (equal); supervision (lead); validation (lead); visualization (lead); writing – original draft (equal); writing – review and editing (equal).

## FUNDING INFORMATION

The funding source had no role in the design and conduct of the study; collection, management, analysis, and interpretation of the data; preparation, review, or approval of the manuscript; and decision to submit the manuscript for publication.

## CONFLICT OF INTEREST STATEMENT

The authors have no conflict of interest to declare.

## Supporting information


Data S1:

## Data Availability

Data is not available unless access request placed through Commission on Cancer.
