# Supplementary material for: Association of patient socioeconomic status with outcomes after palliative treatment for disseminated cancer
Source: Cancer Med. 2024 May 6;13(9):e7028. doi: 10.1002/cam4.7028 (PMC11074703; doi:10.1002/cam4.7028)
Supplement: Supplementary file 1 — Data S1: [file CAM4-13-e7028-s001.docx]

**Supplemental Figure 1.** Consort Diagram


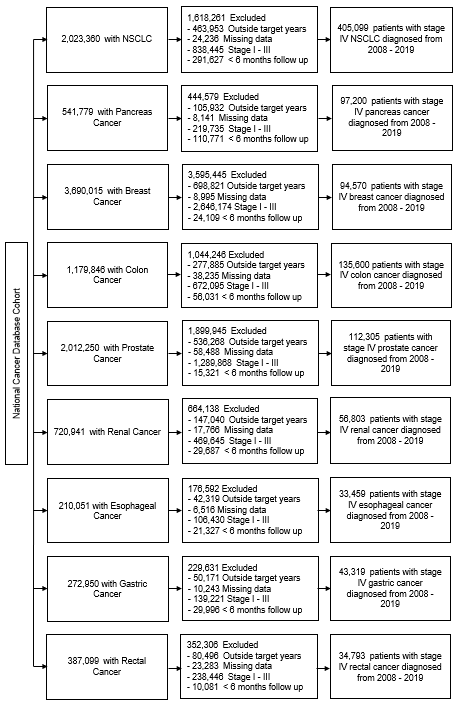


**Supplemental Figure 2.** Forest diagram of Cox proportional hazard models for time from cancer diagnosis to palliative treatment

**
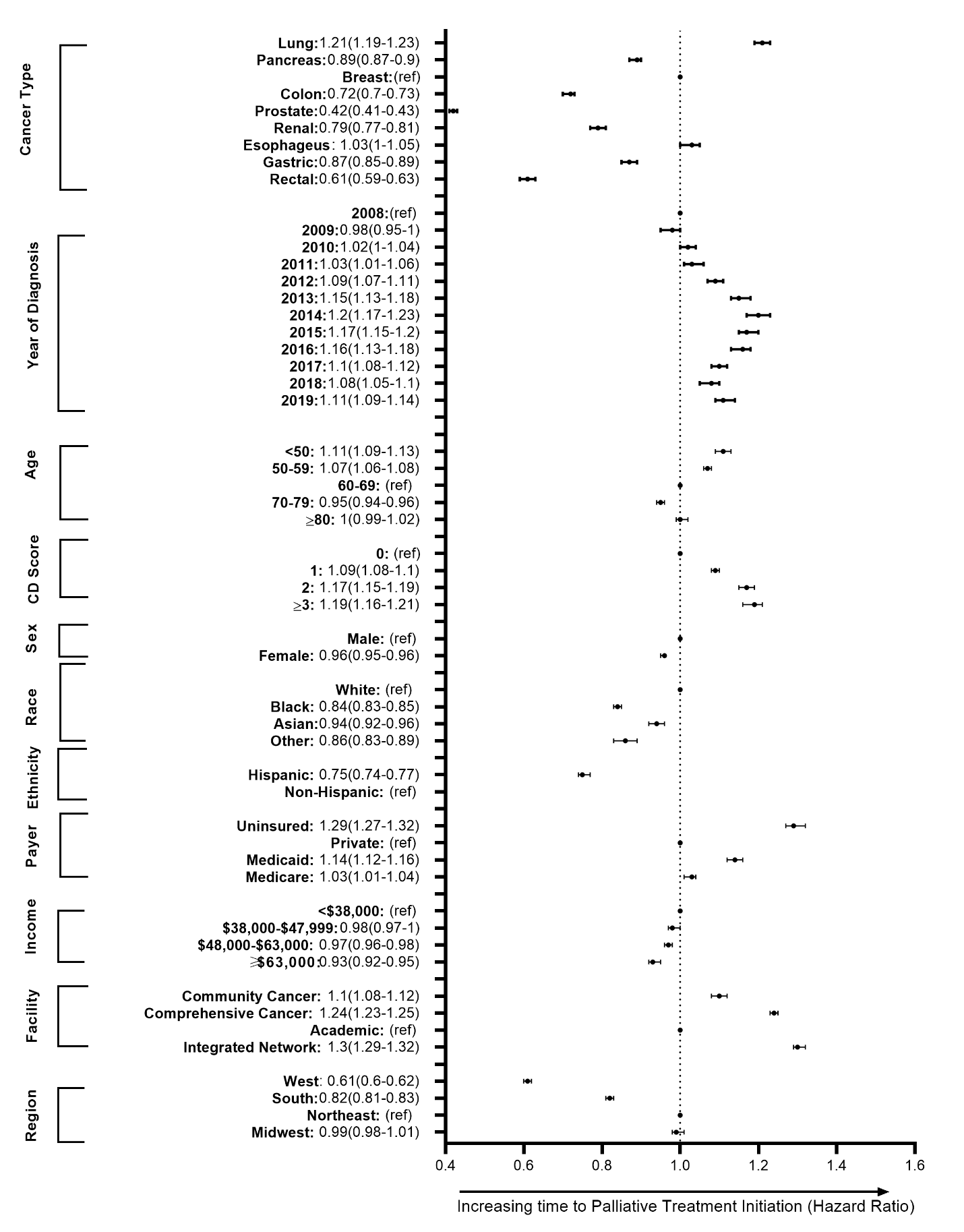
**

**Supplemental Figure 3.** Forest diagram of the logistic regression model for the odds of cancer treatment refusal. (*) Value off scale


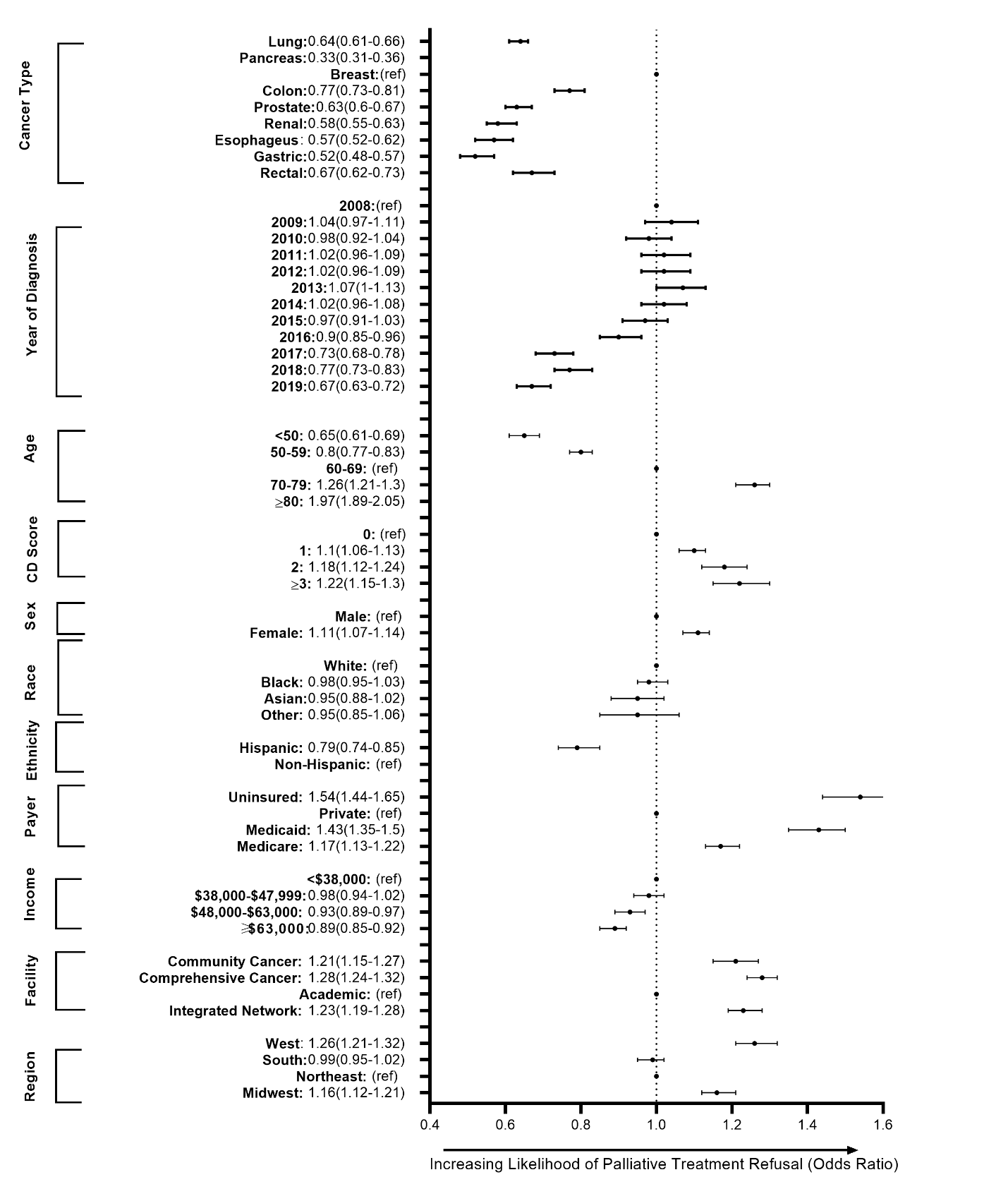


**Supplemental Table 1**: Association between sociodemographic factors and receipt of palliative treatment (full model)

|  | **OR** | **95% Lower** | **95% Upper** | **P value** |
| --- | --- | --- | --- | --- |
| **Cancer Type** |  |  |  |  |
| Breast | Reference |  |  |  |
| Colon | 0.49 | 0.48 | 0.51 | <0.001 |
| Esophagus | 1.31 | 1.28 | 1.35 | <0.001 |
| Gastric | 0.97 | 0.94 | 1.00 | 0.037 |
| Kidney | 0.84 | 0.82 | 0.87 | <0.001 |
| Lung | 1.42 | 1.39 | 1.44 | <0.001 |
| Pancreas | 0.99 | 0.97 | 1.02 | 0.654 |
| Prostate | 0.43 | 0.42 | 0.45 | <0.001 |
| Rectum | 0.73 | 0.71 | 0.75 | <0.001 |
| **Year of Diagnosis** |  |  |  |  |
| 2008 | Reference |  |  |  |
| 2009 | 0.97 | 0.94 | 1.00 | 0.027 |
| 2010 | 1.04 | 1.01 | 1.06 | 0.008 |
| 2011 | 1.06 | 1.04 | 1.09 | <0.001 |
| 2012 | 1.15 | 1.12 | 1.18 | <0.001 |
| 2013 | 1.25 | 1.22 | 1.28 | <0.001 |
| 2014 | 1.34 | 1.30 | 1.37 | <0.001 |
| 2015 | 1.37 | 1.34 | 1.41 | <0.001 |
| 2016 | 1.39 | 1.36 | 1.43 | <0.001 |
| 2017 | 1.35 | 1.31 | 1.38 | <0.001 |
| 2018 | 1.36 | 1.33 | 1.39 | <0.001 |
| 2019 | 1.46 | 1.42 | 1.49 | <0.001 |
| **Age** |  |  |  |  |
| <50 | 1.01 | 0.99 | 1.03 | 0.449 |
| 50–59 | 1.04 | 1.02 | 1.05 | <0.001 |
| 60–69 | Reference |  |  |  |
| 70–79 | 0.98 | 0.97 | 1.00 | 0.011 |
| ³80 | 1.06 | 1.04 | 1.07 | <0.001 |
| **Charlson-Deyo Score** |  |  |  |  |
| 0 | Reference |  |  |  |
| 1 | 1.10 | 1.09 | 1.11 | <0.001 |
| 2 | 1.20 | 1.17 | 1.22 | <0.001 |
| ³3 | 1.23 | 1.21 | 1.26 | <0.001 |
| **Sex** |  |  |  |  |
| Male | Reference |  |  |  |
| Female | 0.95 | 0.94 | 0.96 | <0.001 |
| **Race** |  |  |  |  |
| White | Reference |  |  |  |
| Black | 0.91 | 0.90 | 0.93 | <0.001 |
| Asian | 0.95 | 0.92 | 0.97 | <0.001 |
| Other | 0.84 | 0.81 | 0.87 | <0.001 |
| **Ethnicity** |  |  |  |  |
| Non-Hispanic | Reference |  |  |  |
| Hispanic | 0.79 | 0.77 | 0.81 | <0.001 |
| **Payer Status** |  |  |  |  |
| Uninsured | 1.35 | 1.32 | 1.39 | <.0001 |
| Private | Reference |  |  |  |
| Medicaid | 1.25 | 1.22 | 1.27 | <0.001 |
| Medicare | 1.10 | 1.08 | 1.11 | <0.001 |
| Other Government | 1.21 | 1.17 | 1.26 | <0.001 |
| **Median Income** |  |  |  |  |
| <$38,000 | Reference |  |  |  |
| $38,000-$47,999 | 0.97 | 0.96 | 0.99 | 0.001 |
| $48,000-$63,000 | 0.94 | 0.93 | 0.96 | <0.001 |
| ³$63,000 | 0.86 | 0.85 | 0.88 | <0.001 |
| **Facility Type** |  |  |  |  |
| Community Cancer Program | 1.08 | 1.06 | 1.10 | <0.001 |
| Comprehensive Community Program | 1.17 | 1.16 | 1.19 | <0.001 |
| Academic Cancer Center | Reference |  |  |  |
| Integrated Network Cancer Program | 1.23 | 1.21 | 1.25 | <0.001 |
| **Region** |  |  |  |  |
| Northeast | Reference |  |  |  |
| Midwest | 0.90 | 0.88 | 0.91 | <0.001 |
| South | 0.73 | 0.72 | 0.74 | <0.001 |
| West | 0.55 | 0.54 | 0.56 | <0.001 |

* Predictor remains significant after applying Bonferroni correction for multiple comparisons (p<0.001)

**Supplemental Table 2**. Association between sociodemographic variables and type of palliative treatment modality compared to no treatment (multinomial regression model)

|  | **Surgery** | | **Radiation** | | **Systemic** | | **Pain Management Only** | | **Combination of Modalities** | |
| --- | --- | --- | --- | --- | --- | --- | --- | --- | --- | --- |
|  | OR (95% CI) | p | OR (95% CI) | p | OR (95% CI) | p | OR (95% CI) | p | OR (95% CI) | p |
| **Cancer Site** |  |  |  |  |  |  |  |  |  |  |
| Breast | Reference |  |  |  |  |  |  |  |  |  |
| Colon | 2.05 (1.88-2.25) | <0.001* | 0.09 (0.09-0.1) | <0.001* | 0.78 (0.76-0.81) | <0.001* | 0.87 (0.77-0.97) | 0.013 | 0.43 (0.4-0.45) | <0.001* |
| Esophagus | 1.52 (1.34-1.73) | <0.001* | 1.25 (1.2-1.31) | <0.001* | 1.28 (1.22-1.33) | <0.001* | 1.24 (1.06-1.45) | 0.006 | 1.51 (1.42-1.61) | <0.001* |
| Gastric | 1.9 (1.69-2.13) | <0.001* | 0.53 (0.5-0.56) | <0.001* | 1.32 (1.27-1.38) | <0.001* | 1.38 (1.2-1.58) | <0.001* | 1.04 (0.97-1.11) | 0.277 |
| Kidney | 1.54 (1.37-1.72) | <0.001* | 1 (0.97-1.05) | 0.804 | 0.58 (0.55-0.6) | <0.001* | 1.24 (1.09-1.41) | 0.002 | 1.09 (1.03-1.16) | 0.005 |
| Lung | 1.12 (1.02-1.22) | 0.012 | 1.82 (1.77-1.87) | <0.001* | 0.98 (0.95-1.01) | 0.184 | 1.39 (1.26-1.53) | <0.001* | 1.84 (1.77-1.92) | <0.001* |
| Pancreas | 2.72 (2.48-2.98) | <0.001* | 0.21 (0.2-0.22) | <0.001* | 1.58 (1.53-1.63) | <0.001* | 2.39 (2.15-2.65) | <0.001* | 0.94 (0.89-0.99) | 0.015 |
| Prostate | 0.57 (0.51-0.65) | <0.001* | 0.54 (0.52-0.56) | <0.001* | 0.31 (0.3-0.32) | <0.001* | 0.64 (0.56-0.73) | <0.001* | 0.52 (0.49-0.56) | <0.001* |
| Rectum | 2.66 (2.38-2.97) | <0.001* | 0.45 (0.42-0.47) | <0.001* | 0.83 (0.79-0.87) | <0.001* | 0.75 (0.63-0.9) | 0.001 | 0.9 (0.84-0.97) | 0.006 |
| **Payer Status** |  |  |  |  |  |  |  |  |  |  |
| Private | Reference |  |  |  |  |  |  |  |  |  |
| Uninsured | 1.24 (1.12-1.38) | <0.001* | 1.4 (1.35-1.45) | <0.001* | 1.27 (1.23-1.32) | <0.001* | 2.1 (1.89-2.34) | <0.001* | 1.41 (1.34-1.49) | <0.001* |
| Medicaid | 1.18 (1.09-1.27) | <0.001* | 1.31 (1.27-1.34) | <0.001* | 1.14 (1.11-1.18) | <0.001* | 1.71 (1.58-1.85) | <0.001* | 1.31 (1.26-1.36) | <0.001* |
| Medicare | 1.06 (1-1.12) | 0.046 | 1.08 (1.06-1.11) | <0.001* | 1.08 (1.06-1.11) | <0.001* | 1.28 (1.2-1.36) | <0.001* | 1.14 (1.11-1.18) | <0.001* |
| Other Government | 1.1 (0.92-1.3) | 0.29 | 1.54 (1.46-1.62) | <0.001* | 0.92 (0.86-0.98) | 0.008 | 1.36 (1.12-1.65) | 0.002 | 1.19 (1.09-1.29) | <0.001* |
| **Age** |  |  |  |  |  |  |  |  |  |  |
| <50 | 1 (0.92-1.09) | 0.973 | 0.96 (0.93-0.99) | 0.008 | 0.98 (0.95-1.01) | 0.133 | 1.11 (1.01-1.23) | 0.036 | 1.17 (1.12-1.22) | <0.001* |
| 50–59 | 1.01 (0.96-1.08) | 0.623 | 1.01 (0.99-1.03) | 0.442 | 1.02 (1-1.04) | 0.055 | 1.12 (1.04-1.2) | 0.001 | 1.14 (1.1-1.17) | <0.001* |
| 60-69 | Reference |  |  |  |  |  |  |  |  |  |
| 70–79 | 1.05 (1-1.11) | 0.062 | 1.02 (1-1.04) | 0.033 | 1.01 (0.99-1.03) | 0.483 | 0.93 (0.87-0.99) | 0.031 | 0.84 (0.82-0.87) | <0.001* |
| ≥80 | 1.36 (1.27-1.45) | <0.001* | 1.27 (1.24-1.3) | <0.001* | 0.95 (0.93-0.98) | <0.001* | 1.19 (1.1-1.28) | <0.001* | 0.74 (0.71-0.77) | <0.001* |
| **Sex** |  |  |  |  |  |  |  |  |  |  |
| Male | Reference |  |  |  |  |  |  |  |  |  |
| Female | 1.02 (0.97-1.06) | 0.459 | 0.95 (0.94-0.97) | <0.001* | 0.95 (0.93-0.96) | <0.001* | 0.99 (0.94-1.04) | 0.685 | 0.94 (0.92-0.96) | <0.001* |
| **Year of diagnosis** |  |  |  |  |  |  |  |  |  |  |
| 2008 | Reference |  |  |  |  |  |  |  |  |  |
| 2009 | 0.9 (0.82-0.99) | 0.033 | 0.91 (0.88-0.94) | <0.001* | 1.05 (1.01-1.1) | 0.028 | 0.91 (0.8-1.04) | 0.176 | 1.06 (1-1.14) | 0.065 |
| 2010 | 1.01 (0.92-1.1) | 0.892 | 0.95 (0.92-0.98) | 0.004 | 1.16 (1.11-1.21) | <0.001* | 1.18 (1.04-1.34) | 0.009 | 1.07 (1-1.14) | 0.039 |
| 2011 | 0.83 (0.75-0.91) | <0.001* | 0.95 (0.92-0.99) | 0.009 | 1.28 (1.23-1.34) | <0.001* | 1.03 (0.9-1.17) | 0.7 | 1.13 (1.06-1.2) | <0.001* |
| 2012 | 0.87 (0.79-0.96) | 0.005 | 0.97 (0.93-1) | 0.054 | 1.49 (1.43-1.55) | <0.001* | 1.2 (1.06-1.36) | 0.004 | 1.22 (1.15-1.3) | <0.001* |
| 2013 | 0.86 (0.78-0.95) | 0.002 | 1 (0.97-1.04) | 0.894 | 1.75 (1.68-1.82) | <0.001* | 1.09 (0.96-1.24) | 0.161 | 1.39 (1.31-1.48) | <0.001* |
| 2014 | 0.83 (0.75-0.91) | <0.001* | 1 (0.97-1.04) | 0.968 | 1.97 (1.89-2.06) | <0.001* | 1.27 (1.13-1.44) | <0.001* | 1.54 (1.45-1.63) | <0.001* |
| 2015 | 0.83 (0.75-0.91) | <0.001* | 0.97 (0.94-1) | 0.085 | 2.13 (2.05-2.22) | <0.001* | 1.14 (1.01-1.29) | 0.034 | 1.65 (1.56-1.75) | <0.001* |
| 2016 | 0.76 (0.69-0.84) | <0.001* | 0.92 (0.89-0.95) | <0.001* | 2.15 (2.07-2.24) | <0.001* | 1.29 (1.15-1.46) | <0.001* | 1.91 (1.8-2.02) | <0.001* |
| 2017 | 0.75 (0.68-0.82) | <0.001* | 0.84 (0.81-0.87) | <0.001* | 2.11 (2.03-2.19) | <0.001* | 1.37 (1.22-1.54) | <0.001* | 1.94 (1.83-2.05) | <0.001* |
| 2018 | 0.74 (0.68-0.82) | <0.001* | 0.78 (0.75-0.81) | <0.001* | 2.21 (2.12-2.29) | <0.001* | 1.45 (1.29-1.63) | <0.001* | 2.02 (1.91-2.13) | <0.001* |
| 2019 | 0.79 (0.72-0.87) | <0.001* | 0.8 (0.77-0.83) | <0.001* | 2.37 (2.28-2.47) | <0.001* | 1.55 (1.38-1.74) | <0.001* | 2.33 (2.2-2.46) | <0.001* |
| **Race** |  |  |  |  |  |  |  |  |  |  |
| White | Reference |  |  |  |  |  |  |  |  |  |
| Black | 0.78 (0.73-0.83) | <0.001* | 1 (0.97-1.02) | 0.734 | 0.92 (0.9-0.94) | <0.001* | 0.85 (0.79-0.91) | <0.001* | 0.77 (0.74-0.79) | <0.001* |
| Asian | 1.22 (1.1-1.35) | <0.001* | 0.95 (0.91-0.99) | 0.01 | 0.93 (0.89-0.97) | 0.001* | 0.84 (0.74-0.95) | 0.008 | 0.95 (0.9-1) | 0.053 |
| Other | 0.89 (0.76-1.04) | 0.149 | 0.95 (0.89-1.01) | 0.099 | 0.8 (0.75-0.85) | <0.001* | 0.66 (0.54-0.81) | <0.001* | 0.76 (0.69-0.83) | <0.001* |
| **Ethnicity** |  |  |  |  |  |  |  |  |  |  |
| Non-Hispanic | Reference |  |  |  |  |  |  |  |  |  |
| Hispanic | 0.79 (0.71-0.87) | <0.001* | 0.82 (0.78-0.85) | <0.001* | 0.81 (0.79-0.84) | <0.001* | 0.78 (0.7-0.87) | <0.001* | 0.7 (0.66-0.74) | <0.001* |
| **Median Income** |  |  |  |  |  |  |  |  |  |  |
| <$38,000 | Reference |  |  |  |  |  |  |  |  |  |
| $38,000-$47,999 | 0.94 (0.87-1) | 0.061 | 0.94 (0.92-0.96) | <0.001* | 0.99 (0.98-1.03) | 0.833 | 0.90 (0.84-0.97) | 0.006 | 0.99 (0.95-1.02) | 0.474 |
| $48,000-$63,000 | 0.89 (0.83-0.95) | <0.001* | 0.92 (0.9-0.94) | <0.001* | 0.97 (0.95-0.99) | 0.013 | 0.76 (0.71-0.82) | <0.001* | 0.93 (0.90-0.97) | <0.001* |
| ≥$63,000 | 0.78 (0.73-0.84) | <0.001* | 0.88 (0.86-0.9) | <0.001* | 0.86 (0.84-0.89) | <0.001* | 0.63 (0.59-0.69) | <0.001* | 0.83 (0.80-0.86) | <0.001* |
| **CD score** |  |  |  |  |  |  |  |  |  |  |
| 0 | Reference |  |  |  |  |  |  |  |  |  |
| 1 | 1.31 (1.25-1.37) | <0.001* | 1.06 (1.04-1.08) | <0.001* | 1.07 (1.05-1.08) | <0.001* | 1.39 (1.32-1.47) | <0.001* | 1.13 (1.1-1.16) | <0.001* |
| 2 | 1.45 (1.34-1.56) | <0.001* | 1.14 (1.11-1.17) | <0.001* | 1.15 (1.12-1.19) | <0.001* | 1.69 (1.56-1.83) | <0.001* | 1.25 (1.2-1.3) | <0.001* |
| ≥3 | 1.63 (1.49-1.78) | <0.001* | 1.21 (1.16-1.25) | <0.001* | 1.16 (1.12-1.21) | <0.001* | 1.69 (1.53-1.88) | <0.001* | 1.27 (1.21-1.34) | <0.001* |
| **Facility type** |  |  |  |  |  |  |  |  |  |  |
| Community Program | 0.81 (0.75-0.88) | <0.001* | 1.11 (1.08-1.14) | <0.001* | 1.15 (1.12-1.18) | <0.001* | 0.76 (0.69-0.84) | <0.001* | 1.01 (0.97-1.06) | 0.579 |
| Comprehensive Program | 1 (0.95-1.05) | 0.955 | 1.19 (1.17-1.21) | <0.001* | 1.23 (1.21-1.25) | <0.001* | 0.83 (0.78-0.87) | <0.001* | 1.15 (1.12-1.18) | <0.001* |
| Academic Program | Reference |  |  |  |  |  |  |  |  |  |
| Integrated Network | 1.11 (1.05-1.17) | <0.001* | 1.32 (1.3-1.35) | <0.001* | 1.33 (1.3-1.36) | <0.001* | 0.72 (0.68-0.78) | <0.001* | 0.92 (0.89-0.95) | <0.001* |
| Unknown | 0.66 (0.57-0.76) | <0.001* | 0.73 (0.68-0.78) | <0.001* | 0.78 (0.74-0.83) | <0.001* | 0.51 (0.43-0.61) | <0.001* | 0.84 (0.78-0.92) | <0.001* |
| **Region** |  |  |  |  |  |  |  |  |  |  |
| Northeast | Reference |  |  |  |  |  |  |  |  |  |
| Midwest | 0.7 (0.67-0.74) | <0.001* | 0.9 (0.88-0.92) | <0.001* | 0.94 (0.92-0.96) | <0.001* | 0.48 (0.45-0.52) | <0.001* | 0.95 (0.93-0.98) | 0.002 |
| South | 0.58 (0.55-0.61) | <0.001* | 0.75 (0.74-0.77) | <0.001* | 0.78 (0.77-0.8) | <0.001* | 0.44 (0.41-0.46) | <0.001* | 0.71 (0.69-0.73) | <0.001* |
| West | 0.48 (0.45-0.52) | <0.001* | 0.51 (0.5-0.52) | <0.001* | 0.45 (0.44-0.46) | <0.001* | 0.53 (0.5-0.58) | <0.001* | 0.93 (0.9-0.96) | <0.001* |

* Predictor remains significant after applying Bonferroni correction for multiple comparisons (p<0.001)

**Supplemental Table 3.** Association between sociodemographic factors and receipt of palliative treatment - Breast

|  | **OR** | **95% Lower** | **95% Upper** | **P value** |
| --- | --- | --- | --- | --- |
| **Year of Diagnosis** |  |  |  |  |
| 2008 | Reference |  |  |  |
| 2009 | 0.97 | 0.88 | 1.06 | 0.513 |
| 2010 | 1.11 | 1.01 | 1.21 | 0.027 |
| 2011 | 1.12 | 1.03 | 1.23 | 0.011 |
| 2012 | 1.2 | 1.1 | 1.31 | <.001* |
| 2013 | 1.3 | 1.19 | 1.42 | <.001* |
| 2014 | 1.39 | 1.28 | 1.51 | <.001* |
| 2015 | 1.49 | 1.37 | 1.62 | <.001* |
| 2016 | 1.5 | 1.38 | 1.63 | <.001* |
| 2017 | 1.5 | 1.38 | 1.62 | <.001* |
| 2018 | 1.62 | 1.5 | 1.76 | <.001* |
| 2019 | 1.82 | 1.68 | 1.97 | <.001* |
| **Age** |  |  |  |  |
| <50 | 0.86 | 0.82 | 0.91 | <.001* |
| 50–59 | 1.01 | 0.97 | 1.06 | 0.528 |
| 60–69 | Reference |  |  |  |
| 70–79 | 0.92 | 0.88 | 0.97 | 0.002 |
| ³80 | 0.94 | 0.88 | 0.99 | 0.028 |
| **Charlson-Deyo Score** |  |  |  |  |
| 0 | Reference |  |  |  |
| 1 | 1.09 | 1.04 | 1.14 | <.001* |
| 2 | 1.26 | 1.16 | 1.36 | <.001* |
| 3 | 1.25 | 1.13 | 1.38 | <.001* |
| **Sex** |  |  |  |  |
| Male | Reference |  |  |  |
| Female | 1.04 | 0.92 | 1.18 | 0.540 |
| **Race** |  |  |  |  |
| White | Reference |  |  |  |
| Black | 0.86 | 0.82 | 0.9 | <.001* |
| Asian | 0.91 | 0.83 | 0.99 | 0.034 |
| Other | 0.89 | 0.8 | 1.01 | 0.062 |
| **Ethnicity** |  |  |  |  |
| Hispanic | Reference |  |  |  |
| Non-Hispanic | 0.71 | 0.66 | 0.76 | <.001* |
| **Payer Status** |  |  |  |  |
| Uninsured | 1.4 | 1.3 | 1.51 | <.001* |
| Private | Reference |  |  |  |
| Medicaid | 1.22 | 1.16 | 1.28 | <.001* |
| Medicare | 1.06 | 1.01 | 1.11 | 0.014 |
| Other Government | 1.14 | 0.96 | 1.34 | 0.125 |
| **Median Income** |  |  |  |  |
| <$38,000 | Reference |  |  |  |
| $38,000-$47,999 | 0.97 | 0.93 | 1.03 | 0.328 |
| $48,000-$63,000 | 0.96 | 0.91 | 1.02 | 0.168 |
| ³$63,000 | 0.88 | 0.83 | 0.92 | <.001* |
| **Facility Type** |  |  |  |  |
| Community Cancer Program | 1.26 | 1.2 | 1.32 | <.001* |
| Comprehensive Community Program | 1.07 | 1.03 | 1.12 | <.001* |
| Academic Cancer Center | Reference |  |  |  |
| Integrated Network Cancer Program | 0.78 | 0.72 | 0.85 | <.001* |
| **Region** |  |  |  |  |
| Northeast | Reference |  |  |  |
| Midwest | 0.89 | 0.85 | 0.93 | <.001* |
| South | 0.79 | 0.75 | 0.82 | <.001* |
| West | 0.53 | 0.5 | 0.57 | <.001* |

* Predictor remains significant after applying Bonferroni correction for multiple comparisons (p<0.001)

**Supplemental Table 4.** Association between sociodemographic factors and receipt of palliative treatment - Colon

|  | **OR** | **95% Lower** | **95% Upper** | **P value** |
| --- | --- | --- | --- | --- |
| **Year of Diagnosis** |  |  |  |  |
| 2008 | Reference |  |  |  |
| 2009 | 1.15 | 1.04 | 1.27 | 0.006 |
| 2010 | 1.16 | 1.05 | 1.28 | 0.004 |
| 2011 | 1.21 | 1.1 | 1.34 | <.001* |
| 2012 | 1.32 | 1.2 | 1.45 | <.001* |
| 2013 | 1.67 | 1.53 | 1.83 | <.001* |
| 2014 | 1.81 | 1.66 | 1.98 | <.001* |
| 2015 | 1.86 | 1.7 | 2.04 | <.001* |
| 2016 | 2.05 | 1.88 | 2.23 | <.001* |
| 2017 | 2.22 | 2.04 | 2.42 | <.001* |
| 2018 | 2.42 | 2.22 | 2.63 | <.001* |
| 2019 | 2.64 | 2.43 | 2.88 | <.001* |
| **Age** |  |  |  |  |
| <50 | 0.94 | 0.89 | 1 | 0.062 |
| 50–59 | 1 | 0.95 | 1.05 | 0.953 |
| 60–69 | Reference |  |  |  |
| 70–79 | 1.01 | 0.96 | 1.06 | 0.757 |
| ³80 | 1 | 0.94 | 1.06 | 0.999 |
| **Charlson-Deyo Score** |  |  |  |  |
| 0 | Reference |  |  |  |
| 1 | 1.07 | 1.03 | 1.12 | 0.001* |
| 2 | 1.22 | 1.14 | 1.31 | <.001* |
| ³3 | 1.28 | 1.17 | 1.39 | <.001* |
| **Sex** |  |  |  |  |
| Male | Reference |  |  |  |
| Female | 0.9 | 0.87 | 0.92 | <.001* |
| **Race** |  |  |  |  |
| White | Reference |  |  |  |
| Black | 0.96 | 0.92 | 1.01 | 0.093 |
| Asian | 1.04 | 0.95 | 1.14 | 0.389 |
| Other | 0.82 | 0.72 | 0.94 | 0.003 |
| **Ethnicity** |  |  |  |  |
| Hispanic | Reference |  |  |  |
| Non-Hispanic | 0.88 | 0.82 | 0.94 | <.001* |
| **Payer Status** |  |  |  |  |
| Uninsured | 1.4 | 1.3 | 1.52 | <.001* |
| Private | Reference |  |  |  |
| Medicaid | 1.26 | 1.18 | 1.33 | <.001* |
| Medicare | 1.11 | 1.06 | 1.16 | <.001* |
| Other Government | 1.18 | 1.02 | 1.36 | 0.030 |
| **Median Income** |  |  |  |  |
| <$38,000 | Reference |  |  |  |
| $38,000-$47,999 | 0.96 | 0.91 | 1.02 | 0.162 |
| $48,000-$63,000 | 0.95 | 0.9 | 1 | 0.043 |
| ³$63,000 | 0.87 | 0.83 | 0.92 | <.001* |
| **Facility Type** |  |  |  |  |
| Community Cancer Program | 1.06 | 1 | 1.13 | 0.059 |
| Comprehensive Community Program | 1.1 | 1.06 | 1.14 | <.001* |
| Academic Cancer Center | Reference |  |  |  |
| Integrated Network Cancer Program | 1.13 | 1.07 | 1.18 | <.001* |
| **Region** |  |  |  |  |
| Northeast | Reference |  |  |  |
| Midwest | 0.97 | 0.93 | 1.02 | 0.220 |
| South | 0.79 | 0.76 | 0.83 | <.001* |
| West | 0.59 | 0.55 | 0.63 | <.001* |

* Predictor remains significant after applying Bonferroni correction for multiple comparisons (p<0.001)

**Supplemental Table 5.** Association between sociodemographic factors and receipt of palliative treatment - Esophagus

|  | **OR** | **95% Lower** | **95% Upper** | **P value** |
| --- | --- | --- | --- | --- |
| **Year of Diagnosis** |  |  |  |  |
| 2008 | Reference |  |  |  |
| 2009 | 0.98 | 0.86 | 1.12 | 0.758 |
| 2010 | 1.35 | 1.18 | 1.54 | <.001* |
| 2011 | 1.38 | 1.21 | 1.58 | <.001* |
| 2012 | 1.47 | 1.29 | 1.68 | <.001* |
| 2013 | 1.47 | 1.29 | 1.67 | <.001* |
| 2014 | 1.64 | 1.44 | 1.86 | <.001* |
| 2015 | 1.82 | 1.61 | 2.06 | <.001* |
| 2016 | 1.97 | 1.75 | 2.23 | <.001* |
| 2017 | 1.91 | 1.69 | 2.15 | <.001* |
| 2018 | 1.4 | 1.25 | 1.58 | <.001* |
| 2019 | 1.42 | 1.27 | 1.6 | <.001* |
| **Age** |  |  |  |  |
| <50 | 1.06 | 0.95 | 1.17 | 0.290 |
| 50–59 | 1.04 | 0.97 | 1.11 | 0.279 |
| 60–69 | Reference |  |  |  |
| 70–79 | 1.07 | 1 | 1.14 | 0.060 |
| ³80 | 1.3 | 1.19 | 1.43 | <.001* |
| **Charlson-Deyo Score** |  |  |  |  |
| 0 | Reference |  |  |  |
| 1 | 1.13 | 1.06 | 1.2 | <.001* |
| 2 | 1.21 | 1.09 | 1.34 | <.001* |
| ³3 | 1.5 | 1.32 | 1.71 | <.001* |
| **Sex** |  |  |  |  |
| Male | Reference |  |  |  |
| Female | 0.92 | 0.87 | 0.99 | 0.016 |
| **Race** |  |  |  |  |
| White | Reference |  |  |  |
| Black | 1.02 | 0.94 | 1.12 | 0.593 |
| Asian | 1.11 | 0.94 | 1.32 | 0.211 |
| Other | 0.89 | 0.71 | 1.1 | 0.270 |
| **Ethnicity** |  |  |  |  |
| Hispanic | Reference |  |  |  |
| Non-Hispanic | 0.81 | 0.7 | 0.92 | 0.002 |
| **Payer Status** |  |  |  |  |
| Uninsured | 1.33 | 1.17 | 1.51 | <.001* |
| Private | Reference |  |  |  |
| Medicaid | 1.24 | 1.13 | 1.36 | <.001* |
| Medicare | 1.11 | 1.04 | 1.19 | 0.002 |
| Other Government | 1.2 | 1.02 | 1.42 | 0.032 |
| **Median Income** |  |  |  |  |
| <$38,000 | Reference |  |  |  |
| $38,000-$47,999 | 0.96 | 0.89 | 1.04 | 0.356 |
| $48,000-$63,000 | 0.92 | 0.85 | 0.99 | 0.030 |
| ³$63,000 | 0.84 | 0.78 | 0.91 | <.001* |
| **Facility Type** |  |  |  |  |
| Community Cancer Program | 0.94 | 0.86 | 1.04 | 0.245 |
| Comprehensive Community Program | 1.14 | 1.07 | 1.2 | <.001* |
| Academic Cancer Center | Reference |  |  |  |
| Integrated Network Cancer Program | 1.17 | 1.09 | 1.25 | <.001* |
| **Region** |  |  |  |  |
| Northeast | Reference |  |  |  |
| Midwest | 0.89 | 0.83 | 0.95 | <.001* |
| South | 0.72 | 0.67 | 0.77 | <.001* |
| West | 0.57 | 0.52 | 0.62 | <.001* |

* Predictor remains significant after applying Bonferroni correction for multiple comparisons (p<0.001)

**Supplemental Table 6.** Association between sociodemographic factors and receipt of palliative treatment - Gastric

|  | **OR** | **95% Lower** | **95% Upper** | **P value** |
| --- | --- | --- | --- | --- |
| **Year of Diagnosis** |  |  |  |  |
| 2008 | Reference |  |  |  |
| 2009 | 1.02 | 0.89 | 1.17 | 0.785 |
| 2010 | 1.22 | 1.07 | 1.4 | 0.004 |
| 2011 | 1.16 | 1.01 | 1.32 | 0.035 |
| 2012 | 1.29 | 1.13 | 1.47 | <.001* |
| 2013 | 1.81 | 1.6 | 2.05 | <.001* |
| 2014 | 1.76 | 1.55 | 1.99 | <.001* |
| 2015 | 1.93 | 1.71 | 2.18 | <.001* |
| 2016 | 1.84 | 1.63 | 2.08 | <.001* |
| 2017 | 2.06 | 1.83 | 2.32 | <.001* |
| 2018 | 1.88 | 1.67 | 2.12 | <.001* |
| 2019 | 2.06 | 1.83 | 2.32 | <.001* |
| **Age** |  |  |  |  |
| <50 | 0.98 | 0.9 | 1.07 | 0.641 |
| 50–59 | 1.05 | 0.98 | 1.12 | 0.194 |
| 60–69 | Reference |  |  |  |
| 70–79 | 0.99 | 0.93 | 1.06 | 0.862 |
| ³80 | 1.13 | 1.04 | 1.23 | 0.006 |
| **Charlson-Deyo Score** |  |  |  |  |
| 0 | Reference |  |  |  |
| 1 | 1.16 | 1.09 | 1.23 | <.001* |
| 2 | 1.22 | 1.11 | 1.35 | <.001* |
| ³3 | 1.37 | 1.21 | 1.55 | <.001* |
| **Sex** |  |  |  |  |
| Male | Reference |  |  |  |
| Female | 0.89 | 0.85 | 0.94 | <.001* |
| **Race** |  |  |  |  |
| White | Reference |  |  |  |
| Black | 0.88 | 0.82 | 0.94 | <.001* |
| Asian | 0.98 | 0.88 | 1.08 | 0.620 |
| Other | 0.8 | 0.68 | 0.93 | 0.003 |
| **Ethnicity** |  |  |  |  |
| Hispanic | Reference |  |  |  |
| Non-Hispanic | 0.79 | 0.73 | 0.86 | <.001* |
| **Payer Status** |  |  |  |  |
| Uninsured | 1.24 | 1.11 | 1.39 | <.001* |
| Private | Reference |  |  |  |
| Medicaid | 1.17 | 1.08 | 1.27 | <.001* |
| Medicare | 1.13 | 1.06 | 1.21 | <.001* |
| Other Government | 1.25 | 1.03 | 1.52 | 0.024 |
| **Median Income** |  |  |  |  |
| <$38,000 | Reference |  |  |  |
| $38,000-$47,999 | 1 | 0.93 | 1.08 | 0.978 |
| $48,000-$63,000 | 0.94 | 0.88 | 1.01 | 0.108 |
| ³$63,000 | 0.88 | 0.81 | 0.95 | <.001* |
| **Facility Type** |  |  |  |  |
| Community Cancer Program | 0.96 | 0.87 | 1.06 | 0.413 |
| Comprehensive Community Program | 1.08 | 1.02 | 1.14 | 0.008 |
| Academic Cancer Center | Reference |  |  |  |
| Integrated Network Cancer Program | 1.07 | 1 | 1.15 | 0.040 |
| **Region** |  |  |  |  |
| Northeast | Reference |  |  |  |
| Midwest | 1.01 | 0.94 | 1.08 | 0.835 |
| South | 0.79 | 0.74 | 0.84 | <.001* |
| West | 0.62 | 0.57 | 0.67 | <.001* |

* Predictor remains significant after applying Bonferroni correction for multiple comparisons (p<0.001)

**Supplemental Table 7.** Association between sociodemographic factors and receipt of palliative treatment - Kidney

|  | **OR** | **95% Lower** | **95% Upper** | **P value** |
| --- | --- | --- | --- | --- |
| **Year of Diagnosis** |  |  |  |  |
| 2008 | Reference |  |  |  |
| 2009 | 0.92 | 0.82 | 1.04 | 0.192 |
| 2010 | 1.02 | 0.9 | 1.14 | 0.794 |
| 2011 | 1.1 | 0.98 | 1.23 | 0.107 |
| 2012 | 1.13 | 1.01 | 1.27 | 0.029 |
| 2013 | 1.31 | 1.18 | 1.46 | <.001* |
| 2014 | 1.27 | 1.14 | 1.42 | <.001* |
| 2015 | 1.33 | 1.2 | 1.48 | <.001* |
| 2016 | 1.32 | 1.19 | 1.47 | <.001* |
| 2017 | 1.29 | 1.16 | 1.43 | <.001* |
| 2018 | 1.32 | 1.19 | 1.47 | <.001* |
| 2019 | 1.45 | 1.31 | 1.61 | <.001* |
| **Age** |  |  |  |  |
| <50 | 1.06 | 0.97 | 1.15 | 0.194 |
| 50–59 | 1.01 | 0.95 | 1.07 | 0.870 |
| 60–69 | Reference |  |  |  |
| 70–79 | 0.92 | 0.86 | 0.97 | 0.004 |
| ³80 | 1 | 0.93 | 1.08 | 0.980 |
| **Charlson-Deyo Score** |  |  |  |  |
| 0 | Reference |  |  |  |
| 1 | 1.08 | 1.03 | 1.14 | 0.003 |
| 2 | 1.12 | 1.03 | 1.21 | 0.008 |
| ³3 | 1.11 | 1.01 | 1.23 | 0.039 |
| **Sex** |  |  |  |  |
| Male | Reference |  |  |  |
| Female | 0.98 | 0.94 | 1.03 | 0.387 |
| **Race** |  |  |  |  |
| White | Reference |  |  |  |
| Black | 0.88 | 0.81 | 0.95 | <.001* |
| Asian | 0.96 | 0.84 | 1.1 | 0.540 |
| Other | 0.78 | 0.65 | 0.92 | 0.005 |
| **Ethnicity** |  |  |  |  |
| Non-Hispanic | Reference |  |  |  |
| Hispanic | 0.75 | 0.68 | 0.82 | <.001* |
| **Payer Status** |  |  |  |  |
| Uninsured | 1.45 | 1.3 | 1.62 | <.001* |
| Private | Reference |  |  |  |
| Medicaid | 1.37 | 1.26 | 1.48 | <.001* |
| Medicare | 1.11 | 1.05 | 1.18 | <.001* |
| Other Government | 1.33 | 1.13 | 1.57 | <.001* |
| **Median Income** |  |  |  |  |
| <$38,000 | Reference |  |  |  |
| $38,000-$47,999 | 0.98 | 0.91 | 1.05 | 0.517 |
| $48,000-$63,000 | 0.92 | 0.86 | 0.99 | 0.020 |
| ³$63,000 | 0.87 | 0.82 | 0.94 | <.001* |
| **Facility Type** |  |  |  |  |
| Community Cancer Program | 1.37 | 1.26 | 1.5 | <.001* |
| Comprehensive Community Program | 1.45 | 1.38 | 1.52 | <.001* |
| Academic Cancer Center | Reference |  |  |  |
| Integrated Network Cancer Program | 1.31 | 1.24 | 1.39 | <.001* |
| **Region** |  |  |  |  |
| Northeast | Reference |  |  |  |
| Midwest | 0.86 | 0.81 | 0.91 | <.001* |
| South | 0.66 | 0.62 | 0.7 | <.001* |
| West | 0.54 | 0.5 | 0.58 | <.001* |

* Predictor remains significant after applying Bonferroni correction for multiple comparisons (p<0.001)

**Supplemental Table 8.** Association between sociodemographic factors and receipt of palliative treatment - Lung

|  | **OR** | **95% Lower** | **95% Upper** | **P value** |
| --- | --- | --- | --- | --- |
| **Year of Diagnosis** |  |  |  |  |
| 2008 | Reference |  |  |  |
| 2009 | 0.95 | 0.91 | 0.98 | 0.002 |
| 2010 | 0.97 | 0.94 | 1.01 | 0.145 |
| 2011 | 1.01 | 0.98 | 1.05 | 0.517 |
| 2012 | 1.09 | 1.05 | 1.13 | <.001* |
| 2013 | 1.18 | 1.14 | 1.22 | <.001* |
| 2014 | 1.24 | 1.2 | 1.29 | <.001* |
| 2015 | 1.25 | 1.21 | 1.29 | <.001* |
| 2016 | 1.24 | 1.2 | 1.28 | <.001* |
| 2017 | 1.14 | 1.1 | 1.18 | <.001* |
| 2018 | 1.1 | 1.06 | 1.14 | <.001* |
| 2019 | 1.18 | 1.14 | 1.22 | <.001* |
| **Age** |  |  |  |  |
| <50 | 1.07 | 1.04 | 1.11 | <.001* |
| 50–59 | 1.05 | 1.03 | 1.07 | <.001* |
| 60–69 | Reference |  |  |  |
| 70–79 | 0.96 | 0.94 | 0.98 | <.001* |
| ³80 | 0.99 | 0.97 | 1.02 | 0.498 |
| **Charlson-Deyo Score** |  |  |  |  |
| 0 | Reference |  |  |  |
| 1 | 1.09 | 1.07 | 1.11 | <.001* |
| 2 | 1.15 | 1.12 | 1.18 | <.001* |
| ³3 | 1.19 | 1.15 | 1.23 | <.001* |
| **Sex** |  |  |  |  |
| Male | Reference |  |  |  |
| Female | 0.95 | 0.94 | 0.97 | <.001* |
| **Race** |  |  |  |  |
| White | Reference |  |  |  |
| Black | 0.9 | 0.88 | 0.92 | <.001* |
| Asian | 0.92 | 0.88 | 0.95 | <.001* |
| Other | 0.9 | 0.84 | 0.95 | <.001* |
| **Ethnicity** |  |  |  |  |
| Non-Hispanic | Reference |  |  |  |
| Hispanic | 0.77 | 0.74 | 0.8 | <.001* |
| **Payer Status** |  |  |  |  |
| Uninsured | 1.25 | 1.21 | 1.3 | <.001* |
| Private | Reference |  |  |  |
| Medicaid | 1.18 | 1.15 | 1.21 | <.001* |
| Medicare | 1.07 | 1.05 | 1.09 | <.001* |
| Other Government | 1.2 | 1.14 | 1.27 | <.001* |
| **Median Income** |  |  |  |  |
| <$38,000 | Reference |  |  |  |
| $38,000-$47,999 | 0.96 | 0.94 | 0.99 | 0.001* |
| $48,000-$63,000 | 0.94 | 0.92 | 0.96 | <.001* |
| ³$63,000 | 0.87 | 0.85 | 0.89 | <.001* |
| **Facility Type** |  |  |  |  |
| Community Cancer Program | 1.05 | 1.02 | 1.07 | 0.001* |
| Comprehensive Community Program | 1.15 | 1.13 | 1.17 | <.001* |
| Academic Cancer Center | Reference |  |  |  |
| Integrated Network Cancer Program | 1.24 | 1.22 | 1.26 | <.001* |
| **Region** |  |  |  |  |
| Northeast | Reference |  |  |  |
| Midwest | 0.89 | 0.88 | 0.91 | <.001* |
| South | 0.73 | 0.71 | 0.74 | <.001* |
| West | 0.52 | 0.51 | 0.54 | <.001* |

* Predictor remains significant after applying Bonferroni correction for multiple comparisons (p<0.001)

**Supplemental Table 9.** Association between sociodemographic factors and receipt of palliative treatment - Pancreas

|  | **OR** | **95% Lower** | **95% Upper** | **P value** |
| --- | --- | --- | --- | --- |
| **Year of Diagnosis** |  |  |  |  |
| 2008 | Reference |  |  |  |
| 2009 | 1.06 | 0.97 | 1.16 | 0.215 |
| 2010 | 1.13 | 1.03 | 1.24 | 0.007 |
| 2011 | 1.12 | 1.02 | 1.22 | 0.017 |
| 2012 | 1.15 | 1.05 | 1.26 | 0.002 |
| 2013 | 1.24 | 1.14 | 1.35 | <.001* |
| 2014 | 1.43 | 1.31 | 1.55 | <.001* |
| 2015 | 1.51 | 1.4 | 1.64 | <.001* |
| 2016 | 1.61 | 1.49 | 1.75 | <.001* |
| 2017 | 1.63 | 1.5 | 1.76 | <.001* |
| 2018 | 1.66 | 1.53 | 1.8 | <.001* |
| 2019 | 1.74 | 1.61 | 1.88 | <.001* |
| **Age** |  |  |  |  |
| <50 | 1 | 0.93 | 1.07 | 0.995 |
| 50–59 | 1.01 | 0.96 | 1.06 | 0.701 |
| 60–69 | Reference |  |  |  |
| 70–79 | 1.01 | 0.97 | 1.05 | 0.593 |
| ³80 | 1.06 | 1 | 1.12 | 0.054 |
| **Charlson-Deyo Score** |  |  |  |  |
| 0 | Reference |  |  |  |
| 1 | 1.13 | 1.09 | 1.17 | <.001* |
| 2 | 1.33 | 1.26 | 1.42 | <.001* |
| ³3 | 1.31 | 1.21 | 1.41 | <.001* |
| **Sex** |  |  |  |  |
| Male | Reference |  |  |  |
| Female | 0.99 | 0.96 | 1.02 | 0.669 |
| **Race** |  |  |  |  |
| White | Reference |  |  |  |
| Black | 0.91 | 0.87 | 0.96 | <.001* |
| Asian | 0.96 | 0.87 | 1.05 | 0.368 |
| Other | 0.77 | 0.67 | 0.88 | <.001* |
| **Ethnicity** |  |  |  |  |
| Non-Hispanic | Reference |  |  |  |
| Hispanic | 0.78 | 0.72 | 0.84 | <.001* |
| **Payer Status** |  |  |  |  |
| Uninsured | 1.32 | 1.21 | 1.45 | <.001* |
| Private | Reference |  |  |  |
| Medicaid | 1.29 | 1.21 | 1.38 | <.001* |
| Medicare | 1.13 | 1.09 | 1.18 | <.001* |
| Other Government | 1.19 | 1.04 | 1.36 | 0.012 |
| **Median Income** |  |  |  |  |
| <$38,000 | Reference |  |  |  |
| $38,000-$47,999 | 1.03 | 0.98 | 1.08 | 0.278 |
| $48,000-$63,000 | 0.97 | 0.92 | 1.02 | 0.203 |
| ³$63,000 | 0.85 | 0.81 | 0.9 | <.001* |
| **Facility Type** |  |  |  |  |
| Community Cancer Program | 1.11 | 1.04 | 1.18 | 0.003 |
| Comprehensive Community Program | 1.13 | 1.09 | 1.18 | <.001* |
| Academic Cancer Center | Reference |  |  |  |
| Integrated Network Cancer Program | 1.16 | 1.11 | 1.21 | <.001* |
| **Region** |  |  |  |  |
| Northeast | Reference |  |  |  |
| Midwest | 0.92 | 0.88 | 0.96 | <.001* |
| South | 0.7 | 0.67 | 0.73 | <.001* |
| West | 0.57 | 0.54 | 0.6 | <.001* |

* Predictor remains significant after applying Bonferroni correction for multiple comparisons (p<0.001)

**Supplemental Table 10.** Association between sociodemographic factors and receipt of palliative treatment - Prostate

|  | **OR** | **95% Lower** | **95% Upper** | **P value** |
| --- | --- | --- | --- | --- |
| **Year of Diagnosis** |  |  |  |  |
| 2008 | Reference |  |  |  |
| 2009 | 0.91 | 0.81 | 1.03 | 0.138 |
| 2010 | 1.04 | 0.93 | 1.17 | 0.487 |
| 2011 | 0.98 | 0.87 | 1.1 | 0.752 |
| 2012 | 1.13 | 1.01 | 1.27 | 0.029 |
| 2013 | 1.07 | 0.96 | 1.2 | 0.218 |
| 2014 | 1.19 | 1.07 | 1.33 | 0.001* |
| 2015 | 1.18 | 1.07 | 1.31 | 0.001* |
| 2016 | 1.15 | 1.04 | 1.27 | 0.007 |
| 2017 | 1.07 | 0.97 | 1.19 | 0.170 |
| 2018 | 1.22 | 1.1 | 1.34 | <.001* |
| 2019 | 1.33 | 1.2 | 1.46 | <.001* |
| **Age** |  |  |  |  |
| <50 | 1.3 | 1.15 | 1.48 | <.001* |
| 50–59 | 1.11 | 1.04 | 1.17 | <.001* |
| 60–69 | Reference |  |  |  |
| 70–79 | 1.12 | 1.06 | 1.17 | <.001* |
| ³80 | 1.42 | 1.34 | 1.5 | <.001* |
| **Charlson-Deyo Score** |  |  |  |  |
| 0 | Reference |  |  |  |
| 1 | 1.07 | 1.02 | 1.13 | 0.005 |
| 2 | 1.35 | 1.26 | 1.46 | <.001* |
| ³3 | 1.37 | 1.26 | 1.5 | <.001* |
| **Race** |  |  |  |  |
| White | Reference |  |  |  |
| Black | 0.96 | 0.91 | 1.01 | 0.145 |
| Asian | 0.98 | 0.87 | 1.1 | 0.672 |
| Other | 0.67 | 0.58 | 0.78 | <.001* |
| **Ethnicity** |  |  |  |  |
| Non-Hispanic | Reference |  |  |  |
| Hispanic | 0.9 | 0.83 | 0.98 | 0.018 |
| **Payer Status** |  |  |  |  |
| Uninsured | 1.8 | 1.64 | 1.97 | <.001* |
| Private | Reference |  |  |  |
| Medicaid | 1.67 | 1.55 | 1.8 | <.001* |
| Medicare | 1.2 | 1.14 | 1.26 | <.001* |
| Other Government | 1.38 | 1.2 | 1.59 | <.001* |
| **Median Income** |  |  |  |  |
| <$38,000 | Reference |  |  |  |
| $38,000-$47,999 | 0.96 | 0.91 | 1.03 | 0.258 |
| $48,000-$63,000 | 0.92 | 0.87 | 0.98 | 0.007 |
| ³$63,000 | 0.81 | 0.76 | 0.86 | <.001* |
| **Facility Type** |  |  |  |  |
| Community Cancer Program | 1.31 | 1.22 | 1.41 | <.001* |
| Comprehensive Community Program | 1.47 | 1.41 | 1.54 | <.001* |
| Academic Cancer Center | Reference |  |  |  |
| Integrated Network Cancer Program | 1.38 | 1.31 | 1.45 | <.001* |
| **Region** |  |  |  |  |
| Northeast | Reference |  |  |  |
| Midwest | 0.76 | 0.73 | 0.8 | <.001* |
| South | 0.71 | 0.68 | 0.75 | <.001* |
| West | 0.61 | 0.57 | 0.64 | <.001* |

* Predictor remains significant after applying Bonferroni correction for multiple comparisons (p<0.001)

**Supplemental Table 11.** Association between sociodemographic factors and receipt of palliative treatment- Rectum

|  | **OR** | **95% Lower** | **95% Upper** | **P value** |
| --- | --- | --- | --- | --- |
| **Year of Diagnosis** |  |  |  |  |
| 2008 | Reference |  |  |  |
| 2009 | 0.88 | 0.74 | 1.03 | 0.113 |
| 2010 | 0.87 | 0.74 | 1.03 | 0.101 |
| 2011 | 1 | 0.85 | 1.17 | 0.995 |
| 2012 | 1.18 | 1.01 | 1.38 | 0.034 |
| 2013 | 1.12 | 0.96 | 1.31 | 0.140 |
| 2014 | 1.23 | 1.06 | 1.43 | 0.006 |
| 2015 | 1.33 | 1.15 | 1.54 | <.001* |
| 2016 | 1.46 | 1.26 | 1.68 | <.001* |
| 2017 | 1.46 | 1.27 | 1.68 | <.001* |
| 2018 | 1.68 | 1.46 | 1.94 | <.001* |
| 2019 | 1.69 | 1.47 | 1.95 | <.001* |
| **Age** |  |  |  |  |
| <50 | 0.91 | 0.82 | 1 | 0.051 |
| 50–59 | 1 | 0.92 | 1.08 | 0.983 |
| 60–69 | Reference |  |  |  |
| 70–79 | 1.13 | 1.04 | 1.24 | 0.006 |
| ³80 | 1.64 | 1.48 | 1.82 | <.001* |
| **Charlson-Deyo Score** |  |  |  |  |
| 0 | Reference |  |  |  |
| 1 | 1.12 | 1.04 | 1.21 | 0.004 |
| 2 | 1.19 | 1.04 | 1.37 | 0.011 |
| ³3 | 1.41 | 1.2 | 1.67 | <.001* |
| **Sex** |  |  |  |  |
| Male | Reference |  |  |  |
| Female | 0.99 | 0.93 | 1.04 | 0.637 |
| **Race** |  |  |  |  |
| White | Reference |  |  |  |
| Black | 0.98 | 0.9 | 1.08 | 0.730 |
| Asian | 1.08 | 0.93 | 1.25 | 0.303 |
| Other | 0.8 | 0.63 | 1 | 0.048 |
| **Ethnicity** |  |  |  |  |
| Non-Hispanic | Reference |  |  |  |
| Hispanic | 0.89 | 0.79 | 1.01 | 0.067 |
| **Payer Status** |  |  |  |  |
| Uninsured | 1.59 | 1.41 | 1.78 | <.001* |
| Private | Reference |  |  |  |
| Medicaid | 1.4 | 1.28 | 1.54 | <.001* |
| Medicare | 1.16 | 1.07 | 1.26 | <.001* |
| Other Government | 1.11 | 0.88 | 1.41 | 0.385 |
| **Median Income** |  |  |  |  |
| <$38,000 | Reference |  |  |  |
| $38,000-$47,999 | 0.99 | 0.9 | 1.08 | 0.818 |
| $48,000-$63,000 | 0.98 | 0.9 | 1.07 | 0.648 |
| ³$63,000 | 0.87 | 0.79 | 0.95 | 0.003 |
| **Facility Type** |  |  |  |  |
| Community Cancer Program | 1.13 | 1.01 | 1.26 | 0.037 |
| Comprehensive Community Program | 1.24 | 1.16 | 1.33 | <.001* |
| Academic Cancer Center | Reference |  |  |  |
| Integrated Network Cancer Program | 1.23 | 1.13 | 1.33 | <.001* |
| **Region** |  |  |  |  |
| Northeast | Reference |  |  |  |
| Midwest | 0.93 | 0.86 | 1.01 | 0.079 |
| South | 0.79 | 0.73 | 0.85 | <.001* |
| West | 0.57 | 0.51 | 0.63 | <.001* |

* Predictor remains significant after applying Bonferroni correction for multiple comparisons (p<0.001)

**Supplemental Table 12.** Association between sociodemographic factors and time to palliative treatment

|  | **HR** | **95% Lower** | **95% Upper** | **P value** |
| --- | --- | --- | --- | --- |
| **Cancer Type** |  |  |  |  |
| Breast | Reference |  |  |  |
| Colon | 0.72 | 0.7 | 0.73 | <0.001 |
| Esophageus | 1.03 | 1 | 1.05 | 0.028 |
| Gastric | 0.87 | 0.85 | 0.89 | <0.001 |
| Kidney | 0.79 | 0.77 | 0.81 | <0.001 |
| Lung | 1.21 | 1.19 | 1.23 | <0.001 |
| Pancreas | 0.89 | 0.87 | 0.9 | <0.001 |
| Prostate | 0.42 | 0.41 | 0.43 | <0.001 |
| Rectum | 0.61 | 0.59 | 0.63 | <0.001 |
| **Year of Diagnosis** |  |  |  |  |
| 2008 | Reference |  |  |  |
| 2009 | 0.98 | 0.95 | 1 | 0.048 |
| 2010 | 1.02 | 1 | 1.04 | 0.095 |
| 2011 | 1.03 | 1.01 | 1.06 | 0.007 |
| 2012 | 1.09 | 1.07 | 1.11 | <0.001 |
| 2013 | 1.15 | 1.13 | 1.18 | <0.001 |
| 2014 | 1.2 | 1.17 | 1.23 | <0.001 |
| 2015 | 1.17 | 1.15 | 1.2 | <0.001 |
| 2016 | 1.16 | 1.13 | 1.18 | <0.001 |
| 2017 | 1.1 | 1.08 | 1.12 | <0.001 |
| 2018 | 1.08 | 1.05 | 1.1 | <0.001 |
| 2019 | 1.11 | 1.09 | 1.14 | <0.001 |
| **Age** |  |  |  |  |
| <50 | 1.11 | 1.09 | 1.13 | <0.001 |
| 50–59 | 1.07 | 1.06 | 1.08 | <0.001 |
| 60–69 | Reference |  |  |  |
| 70–79 | 0.95 | 0.94 | 0.96 | <0.001 |
| ³80 | 1 | 0.99 | 1.02 | 0.514 |
| **Charlson-Deyo Score** |  |  |  |  |
| 0 | Reference |  |  |  |
| 1 | 1.09 | 1.08 | 1.1 | <0.001 |
| 2 | 1.17 | 1.15 | 1.19 | <0.001 |
| ³3 | 1.19 | 1.16 | 1.21 | <0.001 |
| **Sex** |  |  |  |  |
| Male | Reference |  |  |  |
| Female | 0.96 | 0.95 | 0.96 | <0.001 |
| **Race** |  |  |  |  |
| White | Reference |  |  |  |
| Black | 0.84 | 0.83 | 0.85 | <0.001 |
| Asian | 0.94 | 0.92 | 0.96 | <0.001 |
| Other | 0.86 | 0.83 | 0.89 | <0.001 |
| **Ethnicity** |  |  |  |  |
| Non-Hispanic | Reference |  |  |  |
| Hispanic | 0.75 | 0.74 | 0.77 | <0.001 |
| **Payer Status** |  |  |  |  |
| Uninsured | 1.29 | 1.27 | 1.32 | <0.001 |
| Private | Reference |  |  |  |
| Medicaid | 1.14 | 1.12 | 1.16 | <0.001 |
| Medicare | 1.03 | 1.01 | 1.04 | <0.001 |
| Other Government | 1.04 | 1.01 | 1.08 | 0.013 |
| **Median Income** |  |  |  |  |
| <$38,000 | Reference |  |  |  |
| $38,000-$47,999 | 0.98 | 0.97 | 1 | 0.009 |
| $48,000-$63,000 | 0.97 | 0.96 | 0.98 | <0.001 |
| ³$63,000 | 0.93 | 0.92 | 0.95 | <0.001 |
| **Facility Type** |  |  |  |  |
| Community Cancer Program | 1.1 | 1.08 | 1.12 | <0.001 |
| Comprehensive Community Program | 1.24 | 1.23 | 1.25 | <0.001 |
| Academic Cancer Center | Reference |  |  |  |
| Integrated Network Cancer Program | 1.3 | 1.29 | 1.32 | <0.001 |
| **Region** |  |  |  |  |
| Northeast | Reference |  |  |  |
| Midwest | 0.99 | 0.98 | 1.01 | 0.326 |
| South | 0.82 | 0.81 | 0.83 | <0.001 |
| West | 0.61 | 0.6 | 0.62 | <0.001 |

* Predictor remains significant after applying Bonferroni correction for multiple comparisons (p<0.001)

**Supplemental Table 13.** Association between sociodemographic factors and patient treatment refusal

|  | **OR** | **95% Lower** | **95% Upper** | **P value** |
| --- | --- | --- | --- | --- |
| **Cancer Type** |  |  |  |  |
| Breast | Reference |  |  |  |
| Colon | 0.77 | 0.73 | 0.81 | <.001* |
| Esophageus | 0.57 | 0.52 | 0.62 |  |
| Gastric | 0.52 | 0.48 | 0.57 | <.001* |
| Kidney | 0.58 | 0.55 | 0.63 | <.001* |
| Lung | 0.64 | 0.61 | 0.66 | <.001* |
| Pancreas | 0.33 | 0.31 | 0.36 | <.001* |
| Prostate | 0.63 | 0.6 | 0.67 | <.001* |
| Rectum | 0.67 | 0.62 | 0.73 | <.001* |
| **Year of Diagnosis** |  |  |  |  |
| 2008 | Reference |  |  |  |
| 2009 | 1.04 | 0.97 | 1.11 | 0.256 |
| 2010 | 0.98 | 0.92 | 1.04 | 0.487 |
| 2011 | 1.02 | 0.96 | 1.09 | 0.498 |
| 2012 | 1.02 | 0.96 | 1.09 | 0.500 |
| 2013 | 1.07 | 1 | 1.13 | 0.043 |
| 2014 | 1.02 | 0.96 | 1.08 | 0.545 |
| 2015 | 0.97 | 0.91 | 1.03 | 0.321 |
| 2016 | 0.9 | 0.85 | 0.96 | 0.002 |
| 2017 | 0.73 | 0.68 | 0.78 | <.001* |
| 2018 | 0.77 | 0.73 | 0.83 | <.001* |
| 2019 | 0.67 | 0.63 | 0.72 | <.001* |
| **Age** |  |  |  |  |
| <50 | 0.65 | 0.61 | 0.69 | <.001* |
| 50–59 | 0.8 | 0.77 | 0.83 | <.001* |
| 60–69 | Reference |  |  |  |
| 70–79 | 1.26 | 1.21 | 1.3 | <.001* |
| ³80 | 1.97 | 1.89 | 2.05 | <.001* |
| **Charlson-Deyo Score** |  |  |  |  |
| 0 | Reference |  |  |  |
| 1 | 1.1 | 1.06 | 1.13 | <.001* |
| 2 | 1.18 | 1.12 | 1.24 | <.001* |
| ³3 | 1.22 | 1.15 | 1.3 | <.001* |
| **Sex** |  |  |  |  |
| Male | Reference |  |  |  |
| Female | 1.11 | 1.07 | 1.14 | <.001* |
| **Race** |  |  |  |  |
| White | Reference |  |  |  |
| Black | 0.98 | 0.95 | 1.03 | 0.444 |
| Asian | 0.95 | 0.88 | 1.02 | 0.134 |
| Other | 0.95 | 0.85 | 1.06 | 0.341 |
| **Ethnicity** |  |  |  |  |
| Non-Hispanic | Reference |  |  |  |
| Hispanic | 0.79 | 0.74 | 0.85 | <.001* |
| **Payer Status** |  |  |  |  |
| Uninsured | 1.54 | 1.44 | 1.65 | <.001* |
| Private | Reference |  |  |  |
| Medicaid | 1.43 | 1.35 | 1.5 | <.001* |
| Medicare | 1.17 | 1.13 | 1.22 | <.001* |
| Other Government | 1.21 | 1.08 | 1.35 | 0.001 |
| **Median Income** |  |  |  |  |
| <$38,000 | Reference |  |  |  |
| $38,000-$47,999 | 0.98 | 0.94 | 1.02 | 0.293 |
| $48,000-$63,000 | 0.93 | 0.89 | 0.97 | 0.001 |
| ³$63,000 | 0.89 | 0.85 | 0.92 | <.001* |
| **Facility Type** |  |  |  |  |
| Community Cancer Program | 1.21 | 1.15 | 1.27 | <.001* |
| Comprehensive Community Program | 1.28 | 1.24 | 1.32 | <.001* |
| Academic Cancer Center | Reference |  |  |  |
| Integrated Network Cancer Program | 1.23 | 1.19 | 1.28 | <.001* |
| **Region** |  |  |  |  |
| Northeast | Reference |  |  |  |
| Midwest | 1.16 | 1.12 | 1.21 | <.001* |
| South | 0.99 | 0.95 | 1.02 | 0.453 |
| West | 1.26 | 1.21 | 1.32 | <.001* |

* Predictor remains significant after applying Bonferroni correction for multiple comparisons (p<0.001)
